# Challenges in Vaccinating Layer Hens against *Salmonella* Typhimurium

**DOI:** 10.3390/vaccines8040696

**Published:** 2020-11-19

**Authors:** Siyuan Jia, Andrea R. McWhorter, Daniel M. Andrews, Gregory J. Underwood, Kapil K. Chousalkar

**Affiliations:** 1 School of Animal and Veterinary Sciences, The University of Adelaide, Roseworthy, SA 5371, Australia; siyuan.jia@adelaide.edu.au (S.J.); andrea.mcwhorter@adelaide.edu.au (A.R.M.); 2 Bioproperties Pty Ltd., Ringwood, VIC 3134, Australia; Dan.Andrews@bioproperties.com.au (D.M.A.); greg.underwood@bioproperties.com.au (G.J.U.)

**Keywords:** *Salmonella* Typhimurium, layer hen, vaccine

## Abstract

*Salmonella* Typhimurium is among the most common causes of bacterial foodborne gastrointestinal disease in humans. Food items containing raw or undercooked eggs are frequently identified during traceback investigation as the source of the bacteria. Layer hens can become persistently infected with *Salmonella* Typhimurium and intermittently shed the bacteria over the course of their productive lifetime. Eggs laid in a contaminated environment are at risk of potential exposure to bacteria. Thus, mitigating the bacterial load on farms aids in the protection of the food supply chain. Layer hen producers use a multifaceted approach for reducing *Salmonella* on farms, including the all-in-all-out management strategy, strict biosecurity, sanitization, and vaccination. The use of live attenuated *Salmonella* vaccines is favored because they elicit a broader host immune response than killed or inactivated vaccines that have been demonstrated to provide cross-protection against multiple serovars. Depending on the vaccine, two to three doses of *Salmonella* Typhimurium vaccines are generally administered to layer hens within the first few weeks. The productive life of a layer hen, however, can exceed 70 weeks and it is unclear whether current vaccination regimens are effective for that extended period. The objective of this review is to highlight layer hen specific challenges that may affect vaccine efficacy.

## 1. Introduction

Foodborne gastrointestinal bacterial pathogens contribute substantially to the total incidence of human diarrheal disease. In particular, non-typhoidal *Salmonella* serovars are among the most commonly identified bacterial species during foodborne gastrointestinal disease outbreaks [1]. Disease symptoms in humans include headache, fever, abdominal cramping, diarrhea, and vomiting. Infection with non-typhoidal *Salmonella* is typically self-limiting, although severe cases may require antimicrobial intervention. Foodborne transmission of *Salmonella* remains a significant public health issue with an estimated 79 million disease cases and 59,000 deaths reported by the World Health Organization in 2010 [1]. Depending on geographical region, the proportion of foodborne salmonellosis cases due to the consumption of animal-sourced foods ranges between 77–89% [2].

Humans can acquire *Salmonella* through the consumption of contaminated eggs, poultry meat, red meat, fruits, and vegetables. Eggs and poultry meat, however, are commonly identified during foodborne outbreaks and point source infections [3,4,5]. The percentage of *Salmonella* cases linked to the consumption of eggs or egg-containing foods varies internationally, but has been found to be 29% in the U.S.A. [6], 20.5% in Europe [7], and 36% in Australia [4]. Raw egg-based sauces such as mayonnaise are among the most common sources, but sweet foods such as custards, mousses, and tiramisu are also often identified as the source of the bacteria during trace back investigation [8].

The genus *Salmonella* is comprised of two species, *Salmonella enterica* and *Salmonella bongeri*. *Salmonella enterica* is further subdivided into six subspecies. It is members of the *Salmonella enterica* subspecies *enterica* (*Salmonella*), however, that are most commonly associated with foodborne disease. Eggs can be contaminated with *Salmonella* through both vertical and horizontal mechanisms. Vertical transmission is most commonly linked with *Salmonella* Enteriditis, and occurs when the bacteria infects the layer hen oviduct, subsequently contaminating the eggs’ internal contents during development [9]. Other serovars, including *Salmonella* Typhimurium, have also been shown to transmit vertically, but less commonly than *Salmonella* Enteriditis [10,11]. Horizontal contamination occurs when an egg comes in contact with a contaminated environment [9]. Bacteria can then adhere to the surface of an egg and migrate into eggshell pores [9] or form biofilms on the surface [12]. A long-term layer hen infection trial demonstrated that horizontal transmission of *Salmonella* Typhimurium is likely to be the most common route of egg contamination [13]. Thus, mitigating *Salmonella* Typhimurium loads on farms can contribute to reducing the bacteria in the food supply chain.

Layer hens can become persistently infected with different non-typhoidal *Salmonella* serotypes [5,14]. The distribution of serotypes, however, is not uniform and exhibits substantial geographic variation [5,14]. Various strains of *Salmonella* Enteritidis and *Salmonella* Typhimurium are most commonly associated with human disease [15]. Thus, mitigating *Salmonella* on farms is an important component in maintaining a safe egg supply chain. In the 1990s, in the U.K., large outbreaks of *Salmonella* Enteriditis resulted in changes in the egg supply chain to control the bacteria, including legislation, food safety education, adopting improved supply chain procedures, as well as on-farm control measures [16]. On-farm controls included the compulsory slaughter of infected parent flocks, decontamination of farms between flocks, treatment of feed, rodent control, strict biosecurity, and the use of vaccination [16]. Combined, these control measures have had an impact reducing the incidence of egg-related cases of salmonellosis caused by *Salmonella* Enteriditis [16].

*Salmonella* Typhimurium remains an important egg-associated pathogen. The use of vaccination as a *Salmonella* control strategy for layer hens is reviewed with a particular focus on current administration procedures. Challenges of achieving efficacious vaccination against *Salmonella* Typhimurium in layer hens are highlighted. On-farm challenges such as the long life of a layer hen potentially necessitating the need for additional vaccine doses are discussed. Aspects of *Salmonella* virulence, intestinal immunity and the involvement of the microbiota are presented. The potential impacts of parasitic infection and maternally derived antibodies on vaccine efficacy are also discussed.

## 2. Vaccination as a *Salmonella* Control Strategy

In egg production systems, vaccination has become an important part of controlling *Salmonella* prevalence [17]. Experimental results have demonstrated that bacteria in the faeces, tissues and eggs of chickens after vaccination was significantly reduced [18]. The existing *Salmonella* vaccines primarily include inactivated, live attenuated, and subunit vaccines [17,19]. In poultry production, vaccines are often used in conjunction with strict biosecurity measures to achieve the best results [20].

## 3. *Salmonella* Typhimurium Vaccines

*Salmonella* is an intracellular pathogen which, after invading the epithelium of the gastro-intestinal tract, enters the gut-associated lymphoid tissue and replicates in macrophages and dendritic cells. There are several commercially available inactivated and live attenuated *Salmonella* Typhimurium vaccines (Table 1). The use of live attenuated vaccines is favoured because they elicit both cell mediated and humoral immune responses [17]. Other vaccine types currently under development include DNA, subunit, and ghost, but are not discussed this review. Vaxsafe^®^ ST (Bioproperties Pty Ltd., Ringwood, Australia), AviPro^®^ Megan^®^ Vac 1 (Pacificvet Limited, Christchurch, New Zealand), and *Salmonella* Typhimurium Δ*cya/crp* are some of the available live attenuated vaccines. The Δ*aroA* and Δ*cya/crp* mutations are the most common mutation for making live attenuated *Salmonella* vaccines. The first *Salmonella* Typhimurium vaccine was generated through the deletion of the metabolic gene, *aroA*, which attenuated the virulence of this strain and conferred protection in mice upon challenge with a wild-type strain [21]. In *aroA* mutants, the Shikimate biochemical pathway is disrupted, preventing the production of essential aromatic amines. An *aroA* mutant vaccine is therefore not able to replicate within the host, reducing the risk of downstream contamination of the food supply chain.

### 3.1. Delivery Methods

Existing live, attenuated *Salmonella* vaccines are administered to poultry in several ways. Coarse spray is a common way to administer vaccines to day-old chicks either in the hatchery or upon placement on the farm. This is an automated method that delivers the vaccine in suspension droplets, which can then attach to the mucosa of the eyes and nasal passages and upper respiratory tract. Gel-based sprays are also used to administer vaccines. This method generates larger droplets which adhere to feathers, enabling the vaccine to be ingested through preening [22]. Preening of feathers post-spray delivers the vaccine to the gastrointestinal tract. *Salmonella* vaccines are also delivered orally via drinking water. Vaccines are diluted into water tanks and distributed through drinker lines. A third route of delivery is intramuscular injection into the pectoralis muscle. Coarse spray and oral inoculation through water are economical, easy to implement and are the most commonly recommended methods of administration. Intramuscular injection requires substantially more labour, but has been linked with a higher circulating antibody response [23,24]. Layer hens commonly receive the first inoculation by coarse spray at hatch. The timing of the administration of the second dose varies amongst manufacturers (between 2–6 weeks of age) but all recommend administration in the drinking water. For layer hens, some vaccine manufacturers recommend a third inoculation in drinking water at 6 weeks of age.

### 3.2. Vaccine Efficacy

Newly hatched chicks are at risk of infection with *Salmonella*, and very low bacterial loads can lead to long-term infections and shedding [25]. Older birds are more resistant to *Salmonella* due to a more developed gut microbiome [26], but the bacteria can colonize the ceca of layer hens and be intermittently shed over their productive lifetime [13,27]. Therefore, due to the long life span of layer hens (75–100 weeks), there is a need to maintain a strong immune response to *Salmonella.* Minimizing *Salmonella* colonization of birds will ultimately reduce bacterial shedding, contributing towards lowering bacterial loads in the shed environment, which will lower the risk of egg contamination.

Few studies have been conducted in layer hens over a commercially relevant period. Results from recent vaccine trials in layer hens have demonstrated that the efficacy of vaccination is limited and does not significantly reduce long term shedding [27,28]. This limited efficacy may be due in part to the inherent immunological resistance of poultry birds to *Salmonella*. Following the first and second doses of an *aroA* mutant *S.* Typhimurium, chicks do not produce a strong IgG response [23,27]. It is not until after the third, intra-muscular dose of vaccine that a significant IgG response is elicited [23,27]. Research conducted using an alternate *aroA* mutant of *Salmonella* Typhimurium initially reduced shedding of the challenge strain but the effect was not persistent upon re-infection [29], indicating that a strong gastrointestinal immune response was not generated. These results are, however, discordant with other international trials testing the vaccine potential of other attenuated mutant strains of *Salmonella* Typhimurium strains [30,31,32]. A survey of the literature revealed a major reason for the low efficacy may be linked with how potential *Salmonella* Typhimurium vaccine strains were prepared. Most trials cultivated vaccine strains in nutritive broth and did not resuscitate the vaccine from lyophilization in diluent prior to oral administration [31,32]. In the poultry production environment, *Salmonella* vaccines are distributed in lyophilized form and reconstituted in an appropriate diluent on-farm.

## 4. Challenges in Vaccinating Layer Hens against *Salmonella* Typhimurium

The variability in outcome of efficacy studies highlights the challenges of vaccinating layer hens against *Salmonella* Typhimurium. On layer farms, residual dust, faeces, and dirt can serve as reservoirs for *Salmonella* and can be responsible for flock-to-flock transmission [33,34,35]. Infection primarily occurs through the oral-faecal route. Rodents, insects, and humans can also transmit *Salmonella* to birds [36,37]. *Salmonella*, however, can circulate in aerosols [38], creating the potential for bacterial attachment to dust particles resulting in inhalation transmission. This complex environment and the extended productive lifespan of a layer hen present unique challenges in achieving effective protection against *Salmonella* Typhimurium infection.

### 4.1. Does Variation in Bacterial Load on the Farm Affect Vaccine Efficacy?

A significant challenge of effectively vaccinating layer hens is the variability in bacterial load that birds could be exposed to on farms. *Salmonella* loads can vary substantially from farm to farm and can be affected by many parameters. During chick rearing, the risk of exposure to *Salmonella* has been shown to be low [39,40]. Following transport to egg production farms (at 15–16 weeks of age), *Salmonella* levels for two free-range flocks were low and remained low over the course of the production period of 78 weeks [39]. A similar investigation of cage production flocks demonstrated that prior to placement of birds in egg production sheds (at 15 weeks of age), *Salmonella* levels were low but increased rapidly following the introduction of point of layer hens [40]. *Salmonella* loads, however, can vary significantly over time [41,42]. Yet, many *Salmonella* Typhimurium vaccine efficacy studies have included the use of very high challenge doses of wild-type bacteria, sometimes as high at 10^8^–10^9^ colony forming units [23,27]. Additionally, layer hen farms are often multi-aged, which means multiple age-classes are housed in the same shed, often in separate areas. The continuous housing of birds, however, inhibits thorough cleaning. Furthermore, older birds can transmit *Salmonella* to younger ones. As such, stringent shed cleaning and the maintenance of biosecurity becomes difficult and the loads of *Salmonella* on-farm are likely to be affected. Thus, it is important to determine the efficacy of vaccination against a range of increasing doses of wild-type *Salmonella* Typhimurium.

### 4.2. Are Current Methods of Vaccine Administration Suitable for Extended Life of a Layer Hen?

Immunogenicity investigations of an *aroA Salmonella* Typhimurium vaccine have shown that following the first two doses of vaccine, chicks develop a poor antibody response [23,27,28]. It is not until after an intra-muscular dose that an antibody response is stimulated in birds [23,27,28]. Similarly, birds administered with an SPI-1 deletion mutant *Salmonella* Typhimurium vaccine strain did not exhibit significant antibodies titres following vaccination, but did following infection with a wild-type strain [43]. The stimulation of an appropriate and effective immune response in chicks is an important component of achieving immunity to *Salmonella* Typhimurium. Thus, future research into alternative diluents potentially containing adjuvants is necessary to enhance the immune response of chicks to vaccination. Furthermore, given the extended lifespan of a layer hen, it is not well established whether the current administration methodologies are sufficient to elicit a protective immune response to continually control *Salmonella* during this period.

### 4.3. Do Unique Aspects of Salmonella Typhimurium Virulence in Poultry Influence Vaccine Efficacy?

The pathogenesis of *Salmonella* Typhimurium in mammals has been studied extensively, but less work has been conducted in poultry. It is clear, however, that the pathogenicity of *Salmonella* Typhimurium is considerably different in chickens. The genome of *Salmonella enterica* is comprised of a number of *Salmonella* pathogenicity islands (SPIs) that contain genes that control bacterial virulence, invasion, dissemination, and persistence [44]. SPI-1, -2, -3, -4, and -5 are the most well characterized. In mice, SPI-1 and SPI-2 are the major bacterial virulence determinants. SPI-1 is responsible for invasion into the intestinal epithelium and is important in intracellular replication, while SPI-2 is essential for survival in macrophages and dissemination of the bacteria [44]. Upon resolution of systemic infection, *Salmonella* is cleared from the liver and spleen and establishes persistent infection in the ceca of hens.

There are a limited number of studies characterizing the role of SPIs in the chicken. Genes in SPI-1 encode proteins that form a type III secretion system (T3SS) that forms a needle complex, enabling the bacteria to invade host cells. SPI-1 encoded proteins are also involved in the modulation of the host immune response during infection, reducing the stimulation of proinflammatory cytokines. In broiler chickens, deletion of SPI-1 reduces *S.* Typhimurium colonization of the ceca [45] and bacterial dissemination to the liver [46] and spleen [45]. Deletion of *spaS*, a major component of the SPI-1 T3SS, has been shown to have little effect in day-old chicks, although in one-week-old birds, the mutant exhibited reduced colonization of the cecum and poor dissemination to the liver [47]. Disruption of the SPI-1 genes *invA*, *invB*, or *invC* attenuated different strains of *Salmonella* Typhimurium but was dependent on the initial virulence of the parent strain [48].

SPI-2 also encodes a T3SS within *Salmonella* vacuoles which disseminates SPI-2 encoded effector proteins that can modulate cellular responses. Disruption of SPI-2 has been found to reduce the capacity of *Salmonella* Typhimurium to disseminate to the spleen [45] and liver [46] of infected chickens but does not affect bacterial colonization of the cecum [45,46]. Deletion of *ssaU*, a component of the SPI-2 T3SS, significantly reduced *Salmonella* Typhimurium colonization of the liver but did not affect intestinal colonization [47]. A library of transposon mutants revealed that disruption of either SPI-1 or SPI-2 in *Salmonella* Typhimurium had little effect on bacterial pathogenesis in chicks [49]. Different strains of *Salmonella* Typhimurium also vary in their virulence in poultry [48]. These variables may contribute to inter-experimental differences. It has also been shown that the expression of SPI-1 and SPI-2 genes is reduced at 42 °C, normal chicken body temperature [50]. At hatch, the body temperature of a chick is ~40 °C but increases over 6 days to ~42 °C, where transcriptional regulators of SPI-1 and SPI-2 exhibit reduced expression [50].

Very limited research has been conducted on the involvement of other SPIs in *Salmonella* Typhimurium pathogenesis in chickens. Transposon mutagenesis has implicated a role for *misL* (SPI-3), but no role for SPI-4, or SPI-5 [49]. *Salmonella* Typhimurium colonizes the cecum of newly hatched chicks and has been shown to upregulate genes in both SPI-3 and SPI-5 [51], but the reason for this is unclear. It should be noted that ablation of SPI-3, SPI-4, and SPI-5 individually was found to have no effect on the pathogenesis of *Salmonella* Enteritidis in poultry but the deletion of one or more of these SPIs affected dissemination to the spleen of infected animals [52]. Further investigation as to the involvement of these SPIs in *Salmonella* Typhimurium virulence is necessary. SPI-6 encodes a type VI secretion system (T6SS) which has also been shown to play a role in the systemic dissemination of *Salmonella* Typhimurium in chickens [53].

The ability of *Salmonella* serovars to invade into the lamina propria of the cecum has been shown to correlate with host immune response to the bacteria [54]. *Salmonella* Infantis, for example, invades only the epithelial layer and produces a weak immune response, whereas *Salmonella* Enteritidis and *Salmonella* Typhimurium are more invasive and penetrate into the basal lateral layer, ultimately stimulating a stronger immune response [54]. The *Salmonella* Typhimurium *aroA* mutant vaccine has been shown to have reduced invasive capacity into the caeca of inoculated chicks [55], but the subsequent immune response was not investigated. In order to stimulate a strong immune response, *Salmonella* bacteria need to be able to invade into the basal lateral layer. Toll-like receptor 5 (TLR5) is located in this region and recognizes bacterial flagellin [56]. Infection with *Salmonella* Typhimurium stimulated the innate immune response in the ceca of one-week-old chicks [57]. In particular, genes encoding toll-like receptors (*TLR1A*, *TLR1B*, *TLR2A*, *TLR4*, *TLR7*, and *TLR15*) and interleukins (IL18, IL1B, IL6, IL8L1, and IL8L2) were among the most highly upregulated [57].

### 4.4. What Role Do Intestinal Immunity and the Gut Microbiota Play in Vaccine Efficacy?

The gastrointestinal system is widely recognized as one of the largest immune organs [58]. IgA secreted from the intestinal mucosa provides a first line of defence against oral exposure to pathogens [59]. The effect of vaccination on IgA levels is somewhat unclear. Secreted mucosal IgA has been shown to be higher in vaccinated-challenged birds compared to challenged-only individuals [60]. In a separate study, however, IgA levels of challenged birds remained higher than vaccinated birds [27]. In mammals, antigen presentation in the Peyer’s patches is a prerequisite for the stimulation of an IgA response. This typically requires movement of bacteria across a specialized epithelium that is not frequently observed in poultry [61]. Thus, the induction of the IgA response in birds likely has a different mechanism. The reduced intestinal invasion of live, attenuated *Salmonella* Typhimurium vaccines may inhibit the development of a specific IgA response, but further research in this area is necessary.

The intestinal specific cellular response to vaccination has not been well characterized in poultry. It has become clear that bacterial enteric infection in mammals induces the activation of a subset of T cells termed Mucosal Associated Invariant T (MAIT) cells [62]. This has been demonstrated for *Salmonella* where MR1, the host-restriction element, controls the activation of MAIT cells through the presentation of bacterially derived vitamin B metabolites [63]. Whether such a pathway is active in chickens is unknown but structural modelling has identified a potential MR1 homologue [64].

The importance of the intestinal microbiota and its role in health and immunity has also become increasingly clear. The intestinal microbiota of chicks develops over time [65]. It has been shown that in the first week, cecal colonization is linked with increased expression of IL-8 and IL-17 [66]. Variation in the composition of the gut microbiota from flock to flock could potentially have effects on the colonization and overall virulence of wild-type *Salmonella* Typhimurium. Furthermore, there is little known about whether live, attenuated bacterial vaccines affect the intestinal microbiota, especially after hatch. To date, there has only been a single study investigating the influence of a *Salmonella* vaccine on the host intestinal microflora [67]. This study found that vaccination of broilers led to changes in the composition of the gut microbiota but the overall relative abundance was not affected [67]. It has been studied that feeding a Bacillus-based probiotic to *Salmonella* Typhimurium-infected chickens reduced the shedding of *Salmonella* Typhimurium in faeces. However, adding probiotics to the feed does not eliminate the bacteria. [68]. Knowledge of the effects that live attenuated *Salmonella* Typhimurium vaccine strains have on the gut microbiota could aid in the optimization of timing of delivery and/or the inclusion of additional doses.

### 4.5. Does Parasitic Infection Impact Vaccine Efficacy?

There is limited evidence on the effects of parasitic infestation on *Salmonella* Typhimurium infection and on vaccination. Poultry can be exposed to several parasites, including *Eimeria* spp., *Cryptosporidium* spp., flagellate parasites, *Blastocystis* spp., and *Ascaridia galli* [69]. Parasite infection can disrupt the host microbiome and impact immunity [70,71,72,73]. Persistent helminth infection, for example, has been shown to alter host susceptibility to microorganisms [74]. In particular, the Th2 immune response induced by helminth infection has been shown to reduce resistance to *Salmonella* infection in mice [72,73,75]. Metabolites produced by parasites in the small intestine of mice can also enhance the virulence and colonization ability of *Salmonella* Typhimurium [76]. It is unclear how parasitic infection would affect *Salmonella* vaccine efficacy in poultry and requires further investigation. *Salmonella* infected *Ascaridia galli* can transmit bacteria to chicks [77].

### 4.6. Do Maternally Derived Antibodies Play a Role?

Maternally derived antibodies (MDAs) in the yolk are the primary source of passive immunity to newly hatched chicks. MDAs have been shown to interfere with vaccination of Newcastle Disease Virus, Marek’s Disease Virus and avian influenza (reviewed in [78]). Although these are all viruses, there is potential for MDAs to interfere with *Salmonella* vaccination. The avian homologue of mammalian IgG is referred to as IgY. IgY antibodies specific for *Salmonella* Typhimurium are present in the yolk and inhibit the growth of bacteria in vitro [79]. Yolk-derived IgY antibodies specific for *Salmonella* Typhimurium inhibit the adherence of bacteria to the human intestinal cell line, Caco2 [80] and significantly improved survival of challenged mice [81]. Where breeder flocks are vaccinated against *Salmonella* Typhimurium with inactivated vaccines, there is vertical transmission of MDAs to progeny chicks. Egg yolk antibodies have been explored as a possible means of passive immunity against *Salmonella* [82], but have not been investigated as an inhibitor of live vaccine colonization after vaccination of pullets. A live attenuated *Salmonella* Typhimurium vaccine strain has been shown to have reduced invasive capacity into intestinal explants of chicks [55] which could have been exacerbated by the present of *Salmonella* specific IgY. Further investigation, however, is needed.

## 5. Conclusions

*Salmonella* Typhimurium represents a significant public health risk due to the potential for contamination in human food supply chains. Layer hens can become persistently infected with *Salmonella* Typhimurium and intermittently shed the bacteria into the farm environment, creating a risk for egg contamination. As part of a multifaceted biosecurity program, vaccination of poultry birds for *Salmonella* has been shown to be effective at contributing to the reduction of bacterial loads in the shed environment [20]. A number of factors, however, can potentially limit the efficacy of vaccination. Inherent management and infrastructural differences from farm to farm can contribute to significant variation in *Salmonella* loads as well as the serovars that may challenge flocks. Additionally, the extended lifetime (75–100 weeks) of a layer hen presents challenges to the maintenance of lasting immunity to *Salmonella* Typhimurium. Increasing vaccine immunogenicity or the administration of additional doses could potentially contribute to providing layer hens extended immunity against *Salmonella*. Future study of the mechanisms enabling *Salmonella* Typhimurium to persist in layer birds will facilitate the design of alternative vaccination regimes to ensure long term efficacy of vaccination. A greater understanding of the role that the gut microbiota have in bird health and limiting *Salmonella* Typhimurium colonization will also be key to improving control of this zoonotic bacteria.

## Figures and Tables

**Table 1:** Live attenuated *Salmonella* Typhimurium vaccines.

Name of Vaccine	Company	Region	Type	Vaccine Program
Vaxsafe^®^ ST	Bioproperties Pty Ltd., Australia	Australia	Live, attenuated vaccine Δ*aroA* mutation	Coarse spray at one-day-old, followed by a booster in the drinking water at 14 days of age.
AviPro^®^ Megan^®^ Vac 1	Elanco Animal Health	U.S.	Live, attenuated vaccine Δ*aroA* mutation	1 day of age—spray; 2 weeks of age—drinking water or spray; 16 weeks of age—drinking water or spray.
Poulvac^®^ ST	Zoetis	U.S.	Live, attenuated vaccine Δ*aroA* mutation	Use at 1 day of age by spray. A second dose should be given at 2 weeks of age in the drinking water.
SALMUNE^®^	Ceva Animal Health	U.S.	Live, attenuated vaccine	Use at one-day-old using coarse spray or drinking water, a second vaccination is required at seven days of age. If chickens are maintained past seven weeks of age, a repeat vaccination is recommended.
*Salmonella* Typhimurium Δ*cya*/*crp*			Live, attenuated vaccine Δ*cya*/*crp* mutation	
